# “They must have seen it, you know.” Body talk, extension talk, and action talk: A qualitative study on how palliative care patients and their significant others express experiencing these nonverbal cues

**DOI:** 10.1371/journal.pone.0299112

**Published:** 2024-04-17

**Authors:** Charlotta Öhrling, Elisabet Sernbo, Inger Benkel, Ulla Molander, Stina Nyblom

**Affiliations:** 1 Palliative Centre, Sahlgrenska University Hospital, Region VGR, Gothenburg, Sweden; 2 Department of social work, University of Gothenburg, Gothenburg, Sweden; 3 Institute of Medicine, Sahlgrenska Academy, University of Gothenburg, Gothenburg, Sweden; The Hong Kong Polytechnic University, HONG KONG

## Abstract

Communication about life-threatening disease and palliative care is essential but often experienced as difficult by those concerned and has mainly been studied in terms of its verbal components. Despite the fundamentality of nonverbal communication, its dimensions in care, especially in the communication by patients and their significant others, has not been as extensively examined. Drawing on a secondary qualitative content analysis of data from 23 interviews—15 with patients in specialized palliative home care in Sweden and 8 with their significant others—this study aims *at* understanding and characterizing how patients verbally express experiences of conveying nonverbal cues about life-threatening disease and its consequences and how their significant others express perceiving these cues. Patients expressed experiences of nonverbal communication in the form of cues conveying meaning about their disease and its consequences, often beyond their control. Whether and how the patients reinforced these cues verbally, depended on individual needs, care for others, and evaluations of relationships. Significant others acknowledged the presence of nonverbal cues and tried to interpret their meaning. Both patients and significant others emphasized the importance of nonverbal cues and actively related to how cues in the form of bodily appearance, aids, objects and acts, serve communicative functions about disease and its consequences. These dimensions of nonverbal communication are characterized as: body talk, extension talk and action talk. This study contributes to an international knowledge base on the complexities of nonverbal communicative aspects in these dimensions and how it affects patients and significant others. Professionals should be aware that dimensions of care, such as prescribed aids, from the patients’ perspective can be perceived as nonverbal cues that might “speak of” disease progression.

## Introduction

Communication represents a basic and vital human need and is arguably particularly important when a person is in a vulnerable position, such as when they have a life-threatening disease [1, 2]. Within healthcare contexts, research has mainly focused on how personnel communicate verbally with patients and their significant others, especially about difficult issues [3–5]. In contrast, nonverbal communication has not received the same attention in the context of care, despite being a proven fundamental and often unavoidable mode of communication central to human interaction [6–11].

From early on, we have an urge to communicate with gestures and continue using, for example, eye contact, mimicking, physical appearance, body movements, or tone of voice in our interaction with others [3, 12]. The close relationship between nonverbal and verbal communication is exemplified by gestures being intrinsically related to language processing [13]. People sense and interpret both verbal and nonverbal expressions in their relationships with others, and when the two forms of expression are consistent, trust, clarity, and rapport increase [7]. Sometimes, nonverbal communication even represents our thoughts and emotions more effectively than verbal language, but they can also occur completely unconsciously and/or subliminally [14, 15].

Some studies discuss nonverbal communication in healthcare contexts, but these primarily relate to communication between professionals and patients, where nonverbal communication has been found to play an important role in whether patients feel satisfied with their dialogue with hospital staff [3, 6, 9]. Even fewer study nonverbal communication related to the patients managing illness/disabilities related to progression of disease, significant others and/or to coping with social interactions; some however discuss how the nonverbal can be used to outline social models of care, and bring together concepts such as stigma and communication in relation to health and illness [16, 17].

However, there seems to be an ongoing discussion about what nonverbal communication is and it is often characterized rather by what it is not, i.e., as not a grammatic structure, without linguistic content, or as not being vocal, verbal, spoken and/or written communication [18–22]. Some definitions of nonverbal communication focus on function rather than on form: on what conveys information or “serve communicative functions” [24] (p 208), on cues that have the potential to communicate meaning [23, 24]. These cues can be defined as “aspects of appearance or nonverbal behavior to which a perceiver may respond or from which they may draw an inference” [7] (p 272). These cues can even occur in absentia, in the form of physical traces and objects [22]. They can also be intentional as well as unintentional, alterable as well as unalterable, such as clothes and make up or height and color of skin [24]. In addition, the physical environment, even in the form of inanimate objects, can contribute to nonverbal communication [25].

Hall et al. further state that there are general knowledge gaps in the field of nonverbal communication, regarding “how cues operate in concert and over time, reflect meaning and intention, and exert their impact” [7] (p 287). This is especially notable regarding the lack of knowledge about nonverbal communication between patients and their significant others, specifically in relation to life-threatening disease and its consequences. Understanding nonverbal communication between patients and their significant others becomes increasingly important, as many patients live longer affected by illness and they, as well as their significant others, must manage the progression of disease in their everyday lives.

This study originates from a previous investigation that aimed to understand verbal communication about life-threatening disease by patients and their significant others [26]. Although not a research question at the time, the analysis of the transcribed interviews revealed that the patients, without having been asked specifically about it, to a large and general extent, talked about physical traces and objects related to their disease and disease progression that nonverbally conveyed information which was perceived to have communicative functions.

The research team therefore initiated a second analysis of the interviews to explore the potential to characterize these experiences, within this verbal data.

Research questions asked concerned what this nonverbally conveyed information was experienced to entail, how these cues could be characterized, and whether patients and significant others expressed similar or different experiences of this nonverbal communication.

The aim of this study is to understand and characterize how patients verbally express experiences of conveying nonverbal cues about life-threatening disease and its consequences, and how their significant others, express perceiving these cues.

## Methods

### Design

A secondary qualitative content analysis of transcribed interviews with patients and significant others was used. The design was underpinned by a hermeneutic approach seeking participants’ experiences and meaning structures to gain a deeper understanding of the experiences expressed by patients’ and significant others [27]. In the hermeneutic position, researchers aim to make sense of how the participants reflect on their own experiences, while simultaneously examining how the researchers’ views and biases might impact the analytical process. Reporting is done according to consolidated criteria for reporting qualitative research [28].

### Participants and settings

Patients enrolled in specialized palliative home care in a part of western Sweden, and significant others of their choosing, were recruited. The significant others could be living together with the patient, or not. Relation to patient can be found in Table 1. All the patients were diagnosed with an incurable, life-threatening disease. Specialized palliative home care included availability to care around the clock from a mobile team consisting of palliative care specialists (physicians and nurses) and daytime access to other professions if required. The patients often remained at home until death. The settings for the interviews were arranged in accordance with the participants’ wishes, in most cases their home environment. Interviews were conducted face to face or, when preferred by the participant, over the telephone.

**Table 1:** Demographic and diagnostic data.

	Patients (n = 15)	Significant others (n = 8)
**Gender**		
Female	9	4
Male	6	4
**Age (year)**		
20–40	0	3
41–60	2	1
61–80	9	4
81—	4	0
**Living**		
Solo	7	0
Accompanied	8	8
**Employment status**		
Working	0	4
On sick leave	3	0
Retired No answer	12	3 1
**Highest level of education**		
Elementary school	1	0
High school	8	2
College/University	6	5
No answer	0	1
**Patient’s disease**		
Cancer*	14	7
ALS	1	1
**Patient’s disease duration (yr)**		
<1	2	3
1–2	4	2
3–5	2	1
6–10	3	1
>10	4	1
**Relation to patient**		
Spouse, partner	-	3
Child	-	3
Relative	-	1
Friend	-	1

*liver-, colon-, breast-, lung-, ovarian-, CNS-, pancreatic-, bile duct cancer

Of the 30 patients who met the study criteria and were approached, 18 gave their consent to participate. Before their scheduled interviews, there were three dropouts due to being too tired and death. Interviews were conducted with 15 patients (P). Of the 15 significant others eligible to participate, nine gave their consent. One could not be reached. Interviews were conducted with eight significant others (S). In total, 23 interviews were conducted. Demographic and diagnostic data are presented in Table 1

### Sample and recruitment

A consecutive sampling method was used within the group of eligible patients. The plan was to include 20 patients and 20 significant others. The physicians responsible for the patients were informed about the study and asked to give written and verbal information about the study to eligible patients. Patients willing to participate sent their written consent to the research team by post and were contacted afterwards by telephone. The patients were asked whether they had significant others and if they consented to them being informed about the study. With the patients’ permission, their significant others were given written information about the study and asked to send written consent if they were interested in participating. The inclusion criteria were: being 18 years or older, understanding and speaking Swedish, and having the capacity to provide informed consent and the perceived energy to participate in an interview. The exclusion criteria were: not fulfilling all the inclusion criteria and not wanting to participate in the study.

### Data collection

The present study is a secondary analysis of data collected through in-depth semi-structured interviews with patients and significant others. Interviews were digitally recorded and professionally transcribed verbatim. No field notes were made. Data was stored on a password-protected usb. Audio recordings, transcribed interviews and background factors were anonymized and marked consecutively with a number and could not be linked to the consent form.

Transcripts were not returned to participants for comment. The average length of the interviews was 43 and 32 minutes for patients and significant others respectively. No repeat interviews were performed. Data were collected between October 2019 and April 2020.Furthermore, the data were rich, presumably due to the interviews being performed by social workers trained in interviewing. The interviewers were unknown to the participants and not involved in their care. Physicians and/or nurses were not present during the interviews.

### Data analysis

In this secondary analysis, all the transcripts were re-read by the individual researchers to identify examples of nonverbal cues. Qualitative content analysis was used to find the meaning of the participants’ expressions [29, 30]. No software was used. All the authors individually marked text relevant to the research questions and confirmed these later in a joint discussion. By further analysis, units of meaning were identified and grouped into themes describing phenomena and overall meaning in the participants’ interview data [31]. Analysis was performed with the cooperation of all the authors to interpret the results. Any discrepancies were discussed until consensus was reached, no further themes were identified, and saturation was reached. All the authors were experienced in qualitative analysis.

### Ethical issues

This study was approved by the Swedish Ethical Review Authority (No. 2019–03082; date of approval: 05/06/2019). Ethical considerations have been made throughout the study with consent ensured regularly, and with regard to the illness and the patients (and their significant others) being tired. Inspired by Tracy, we aimed for sincerity, credibility, and resonance in relation to the participants’ stories [32]. During the interviews, this was primarily concretized in efforts to be sensitive to situational and relational ethics. The analysis encouraged honesty and transparency about differences in reflections of the researchers related to the research team members’ different professional and theoretical backgrounds in social and medical sciences. We further strived to offer in-depth illustrations and include multiple and varied voices in the writing. By achieving saturation, value was also given to supporting transferability of the results and underlining the study’s potential to be valuable to questions of nonverbal communication across a variety of contexts, especially in healthcare settings.

## Results

When patients were asked about experiences of telling others about their illness, they clearly expressed nonverbal dimensions of this communication, in the form of different cues. The significant others were also asked about their experiences with communication and reported that they noticed nonverbal cues.

Three themes, characterizing these cues, common to patients and significant others, were identified: *Body talk*, *Extension talk* and *Action talk*. The first two themes describe the nonverbal communication of objects—how the patient´s body changes and how its extensions “talk”. The third theme focuses on how cues in certain kinds of actions entail nonverbal communication. A fourth theme, *To say*, *or not to say*?, centers around if and how the nonverbal is verbally communicated by the patient, and a fifth theme, *Significant others*’ *understanding*, focuses specifically on the experiences of significant others.

### Body talk–changes in the patient´s bodily appearance

This theme is centered around changes in bodily appearance, as the body no longer looks as expected or as it did before. In different ways, the body has become affected by disease and continues to be affected as the illness progresses. Weight loss, in particular, was described by the patients as an apparent nonverbal cue: body talk. Patients expressed the conviction that significant others related to the disease just by looking at the body and that it was hard for the patient to conceal disease even if they wanted to:

“*But now*, *you cannot lie*. *Now*, *even an idiot can see that I am ill*.*”* (P2)

Significant others also acknowledged changes in bodily appearance as nonverbal signs of illness. Like the patients, significant others perceived weight loss and fatigue, for example, as nonverbal cues:

“*Every time we see our friend*, *he is paler and thinner and more tired*.*”* (S7)

Worsening of these signs, and/or the appearance of new signs, indicated that the patient was becoming sicker. This was expressed as particularly apparent to significant others in retrospect, noticing changes over time.

### Extension talk–the body´s need for assistance

As the illness and its effects on the body progressed, aids were needed to assist the body in the routines of daily life. It became increasingly apparent to both patients and significant others that the body could no longer uphold its functions the way it did before. Aids, such as wheelchairs, walkers, and adjustable beds, made an extension of the body possible by replacing functions that had been lost. These prolongations of the body could take the form of material objects attached to the body, such as a drip equipment or an oxygen hose. It could also entail objects that were not physically attached to the body, but still indicated the presence of disease and the body’s need for assistance. Examples mentioned were packages of pharmaceuticals and incontinence aids placed in the patient’s home, as well as visiting cars and personnel from the mobile team.

“*Then I sat there on a small stool waiting for the flex-line* [medical transport] *and the neighbor came and said*, *‘Are you sick*?*’ It made a big difference to her and she said so several times*.*”* (P7)

From this quote, we saw that objects, such as the body on the stool and the medical transport, “spoke” nonverbally to the neighbor, who responded by posing a verbally communicated question to the patient. Objects, even (in) the hands of others, can thus be characterized as constituting nonverbal cues: extension talk.

### Action talk—what one does or does not do

In addition to the body and its extensions, both patients and significant others stated that actions, as in what one does or does not do, were perceived as nonverbal cues: action talk.

“*She’s carrying on fighting right now by making lists of things to fix*, *and it’s almost daily … she’s cleaning*, *death cleaning in various ways with banks and whatnot*.*”* (S2)

In the quote above, the action was new and related to the illness, its progress, and the perceived future. Actions could also talk when related to activities that the patient could no longer perform because of fatigue or movement constraints. These changes, brought on by disease, were described as tangible signs, cues, of illness that make the need for assistance in daily life apparent:

“*I couldn’t bear to go up the stairs*. *After two or three steps I had to sit down*… *they must have seen it*, *you know*… *The worst part was that I couldn’t be of any help*… *my wife had to do everything*.*”* (P5)

When significant others took over the execution of activities that the patients normally performed but were no longer able to do due to illness, this could also be understood as nonverbal cues:

“*She asked me to change a pressure sore dressing today*. *I absolutely can do that*. *And then I changed it and saw that she had a big flesh wound all over her lower back*. *You don’t see that until you do it*. *I think she says certain things and you understand certain things; it depends a bit on the circumstances at the time*.*”* (S4)

In situations where significant others assisted the patients, new and perhaps unexpected forms of both body talk and extension talk might become apparent to the significant others and communicate different things about the disease itself and/or the future. The significant others expressed understanding of these situations as a specific form of nonverbal cues, depending on the action and the information conveyed in that action. This relational aspect is further developed in the two following themes in which patients and significant others are discussed separately.

### To say, or not to say?

In the interviews, patients described how they sometimes spoke about what they felt was apparent in the form of nonverbal cues, such as an oxygen hose, and sometimes they did not. Occasionally, they also verbally communicated what the hose meant in relation to the progression of the disease and its prognosis.

The patients gave different reasons for verbally commenting on what was expressed nonverbally. One intention was to make room for the patients’ own interpretations and understanding of the situation. Another intention was to avoid others speculating:

“*And then she said*: *‘you have lost a lot of weight*.*’ And then I thought*, *what the heck*, *I don’t need to pretend here*, *I’ll tell it like it is*.*”* (P15)

Another approach was not to comment on what you know others could see and instead rely on the nonverbal cues as being obvious and thus sufficient. Others said they refrained from verbal comments as they did not want others to know. This was related, in particular, to more peripheral relationships and/or to experiencing the condition as embarrassing. Yet another reason could be that the patient wanted to protect others as well as themselves.

The experienced good quality of a relationship may mean that the patients verbally comment to gain support and/or to help significant others to interpret nonverbal cues in the way the patients want them to. However, good quality may also mean that the patient does not verbally comment in order to avoid burdening the significant others:

“*She* [friend] *is fragile*, *and I can see that she feels bad seeing me feel bad*. *Yes* [pause], *I have to try to* [pause], *yes*, *I* [pause], *we* [pause], *you don’t talk about that*, *you talk about other things*.*”* (P10)

In several ways, the patients actively related to how the body and its extensions talk. If and how this was verbally commented on depended on the patients’ intentions, as well as of the reactions (actual and expected) of others.

Understanding communication through aspects of personal relationships was also discussed by the significant others.

### Significant others’ understanding

Significant others discussed nonverbal cues less than the patients did. We do not know why, as this was a secondary data analysis, and none of the participants were specifically asked about nonverbal communication. However, they did say they noticed, tried to interpret, and considered nonverbal cues:

“*We’re doing everything we can*, *but we’re giving half morphine and all the painkillers and stuff we can*, *but is she dying or isn’t she dying*?*”* (S4)

The significant others expressed that they understood the progressive nature of the disease when nonverbal cues were combined with certain forms of verbal communication. This was the case when, for example, the patient told the significant others that the treatment had resulted in new consequences, that treatment plans were changed, or when a professional had recommended more advanced care at home or at a hospice. Related to nonverbal cues, the verbal communication was interpreted as containing more information than the words actually spoken. Thus, from the bystander’s position, significant others described trying to combine and interpret these different forms of communication in a way that made sense to them:

“… *they were in agreement* that *we should have help at home*, *because he needed that much care*. *Blood and discharges of fluid and so much more*.*”* (S3)

However, the significant others rarely reported commenting on nonverbal cues directly to the patient. Rather, they tried to understand, even if this was experienced as difficult.

## Discussion

This qualitative study identifies the importance of nonverbal communication, specifically about life-threatening disease focusing on patients in palliative care.

Nonverbal communication can be described as a behavior without linguistic content including cues, added information that is seen, and similar to cues that can been heard in verbal communication, such as tone of voice [6, 7, 33]. Nonverbal communication can thus be understood in relation to that which conveys information, a function, rather than a form.

This study shows that such cues, specifically related to the progression of disease, can include changes in the patient’s bodily appearance, the sudden existence of an oxygen hose, the need for assistance in daily living, or wounds that become visible when a significant other helps change bandages. Physical appearance, in the form of unintentional, less alterable, characteristics such as skin color or height, can be regarded as nonverbal cues [23, 34]. As shown in this study, unintentional changes in bodily appearance caused by serious disease, for example, very low weight (cachexia) and change of skin color (jaundice), also constitute nonverbal cues.

Nonverbal communication can be understood as sometimes being an effect of disease beyond the control of the patient (or significant others) [3, 33]. Changes in the body become apparent to others, regardless of what the patient wants others to know. While objects, like e.g. aids in the form of wheelchairs, make extensions of the body possible and enable patients to do more things by themselves, they also carry nonverbal communication beyond the patients’ control. They function as cues that convey meaning to which a perceiver may respond or draw an inference [7, 23, 24]. Unlike clothing, which is a common extension of the body and an optional form of nonverbal communication, extensions in the context of care are often of vital importance, without realistic alternatives [35, 36].

The study contributes to broaden knowledge in the field of nonverbal communication, especially in relation to what Hall et all describe as a knowledge gap regarding “how cues operate in concert and over time, reflect meaning and intention, and exert their impact” [7] (p 287). The findings add to the understanding of nonverbal communication about life-threatening disease and its consequences, specifically regarding how different nonverbal cues can be characterized as: body talk, extension talk, and action talk. These dimensions can be used to understand what nonverbal communication can entail, rather than describe what nonverbal communication is not. This study focuses on palliative care, but it is probable that the described dimensions of nonverbal cues can be valuable also in a broader context of healthcare where the nonverbal can be understood as “spoken” by the body itself, as well as by artifacts and actions.

The study underpins the importance of increased awareness about the significance and complexities of nonverbal communicative aspects embedded in palliative care. On the one hand, professionals need to consider the communicative aspects of their professional practice, such as when prescribing aids and medicines. These artifacts constitute extension talk that affects both patients and significant others in more ways than any of those involved might intend. A wheelchair helps the patient move, but as an extension of the body, it also talks about the progression of the disease. So does the visibility of a visit from the mobile team, regardless the intentions of the professionals.

Furthermore, in relation to communication between patients and significant others, professionals need to be aware of the considerations of those involved and the complexities in these considerations. Whether and how the patients verbally reinforce this nonverbal communication depends on individual intentions, such as not wanting to discuss their situation with others or wanting to avoid speculation and/or experiencing feelings of relief when telling it like it is. The patients’ verbal communication is also related to concern for others and evaluations of relationships. Motives for choosing whether to verbally comment can therefore be understood as complex, and related to ideas about how the perceiver may respond to, or draw inference from, the nonverbal cues.

Some patients also express experiencing nonverbal cues as sufficient communicators in themselves or expecting them to be so. However, significant others do not express this feeling of the nonverbal as sufficient. Instead, they express trying to interpret nonverbal cues from the patient (as well as from the healthcare professionals) and try to piece together the puzzle, often without verbally commenting on this directly to the patient. The patients and their significant others can in other words be understood to agree on the cues conveying information, but not always on how to draw inference on them [7].

The results of this study should however not be interpreted as a need for professionals to always try to correct these inconsistencies. For instance, there might be inconsistencies between nonverbal and verbal communication by patients and others, such as when patients choose not to reinforce verbally the nonverbal, but this can be understood as in line with the patient’s intentions related to individual and/or interactional considerations, rather than as a problem professionals need to solve.

## Strengths and limitations

A strength of the study was that all the patients were diagnosed with an incurable, life-threatening disease, mostly cancer, and were thus representative of patients in specialized palliative care in Sweden. This may increase transferability to similar contexts while limiting it to others. While we could not rule out bias among the physicians who selected the patients, the aforementioned representativeness among the patients included speaking against bias. Although we experienced recruitment challenges due to the COVID-19 pandemic, gender distribution was fairly even, and sample sizes were considered adequate, as saturation in the analysis was reached. As this was a second analysis of transcribed verbal data, nonverbal communication is limited to what is described through verbal expressions by the participants.

Most of the participants were highly educated, which may have influenced the results if, for example, they could more easily express themselves verbally. All the participants also spoke Swedish, and non-Swedish speaking persons may have given other perspectives on the research questions, which also applied to those invited who chose not to participate.

## Conclusion

Nonverbal communication is an important form of communication that exists in itself. With regard to life-threatening disease and its consequences, it has been found to entail cues that can be characterized in three dimensions: body talk, extension talk, and action talk. Nonverbal communication is embedded in relationships between patients and significant others. It is also embedded in objects and acts, and not least related to treatment, such as in aids that are designed to facilitate but, as nonverbal cues they also convey information, they talk. So does a visit from professionals, regardless of their intentions. Whether and how nonverbal cues are reinforced verbally depends on individual needs, concern for others, as well as an evaluation of the relationship.

With respect to the significance, complexities, and potential of nonverbal communication, especially in palliative care, further knowledge and education are essential for professionals.

Two particularly important themes for future research emerged: first, to theoretically analyze the described dimensions, complexities, and importance/meaning of nonverbal communication outlined in this study in more depth and, second, to examine the extent to which nonverbal cues and communication could be used by professionals to verbalize difficult issues, such as disease progression, in their communication with patients and significant others.
